# Genome-wide characterization of the *SHORT INTER-NODES/STYLISH* and *Shi-Related Sequence* family in *Gossypium hirsutum* and functional identification of *GhSRS21* under salt stress

**DOI:** 10.3389/fpls.2022.1078083

**Published:** 2023-01-04

**Authors:** Chendong Sun, Li Yu, Shuojun Zhang, Qijuan Gu, Mei Wang

**Affiliations:** ^1^ Institute of Horticulture, Zhejiang Academy of Agricultural Sciences, Hangzhou, China; ^2^ Institute of Crop Science, College of Agriculture and Biotechnology, Zhejiang University, Hangzhou, China; ^3^ Key Laboratory of Microbiol Technology and Bioinformatics of Zhejiang Province, Zhejiang Institute of Microbiology, Hangzhou, China

**Keywords:** genome-wide characterization, *SRS* family, *Gossypium hirsutum*, salt stress, regulation of gene expression

## Abstract

Saline stress is a significant factor that caused crop growth inhibition and yield decline. SHORT INTERNODES/STYLISH (SHI/STY) and SHI-RELATED SEQUENCE (SRS) transcription factors are specific to plants and share a conserved RING-like zinc-finger domain (CX_2_CX_7_CX_4_CX_2_C_2_X_6_C). However, the functions of *SHI*/*STY* and *SRS* genes in cotton responses to salt stress remain unclear. In this study, 26 *GhSRSs* were identified in *Gossypium hirsutum*, which further divided into three subgroups. Phylogenetic analysis of 88 *SRSs* from8 plant species revealed independent evolutionary pattern in some of *SRSs* derived from monocots. Conserved domain and subcellular location predication of GhSRSs suggested all of them only contained the conserved RING-like zinc-finger domain (DUF702) domain and belonged to nucleus-localized transcription factors except for the GhSRS22. Furthermore, synteny analysis showed structural variation on chromosomes during the process of cotton polyploidization. Subsequently, expression patterns of *GhSRS* family members in response to salt and drought stress were analyzed in *G. hirsutum* and identified a salt stress-inducible gene *GhSRS21*. The GhSRS21 was proved to localize in the nuclear and silencing it in *G. hirsutum* increased the cotton resistance to salt using the virus-induced gene silencing (VIGS) system. Finally, our transcriptomic data revealed that *GhSRS21* negatively controlled cotton salt tolerance by regulating the balance between ROS production and scavenging. These results will increase our understanding of the *SRS* gene family in cotton and provide the candidate resistant gene for cotton breeding.

## 1 Introduction

*SHI*/*STY* and *SRS* family members, a plant-specific transcription factors, are defined by the presence of a conserved RING-like zinc-finger domain (CX_2_CX_7_CX_4_CX_2_C_2_X_6_C), the vast majority of which also contain the IXGH domain (Kuusk et al., 2006; Zhao et al., 2020). The RING finger domain is believed to confer E3 ubiquitin ligase activity and mediate the ubiquitination and proteasome-dependent degradation of target protein or confer the DNA and RNA binding activity (Fridborg et al., 2001; Zhao et al., 2020). The IXGH domain is rich in acidic amino acid residues, which are considered as transcriptional activators (Singh et al., 2020).

It has been shown that SRS transcription factors are involved in gibberellin (GA) and auxin signaling pathways (Fridborg et al., 1999; Fridborg et al., 2001; Singh et al., 2020; Yuan et al., 2020) and diverse growth and development processes in plant such as lateral root development and floral organ morphogenesis (Kuusk et al., 2002; Sohlberg et al., 2006; Staldal et al., 2012; Singh et al., 2020; Yuan et al., 2020). In *Arabidopsis*, there are 9 described members in the SHI/STY and SRS family (Eklund et al., 2011; Zhao et al., 2020). The first *SHORT INTERNODES/STYLISH* (*SHI*/*STY*) and *SRS* family gene to be identified was *AtSHI* and its transposon insertion mutants displayed a dwarf phenotype similar to the mutants defective in the biosynthesis of gibberellin (Fridborg et al., 1999). However, the application of GA could not rescue the phenotype the dwarf phenotype of *atshi*, indicating the potential role of AtSHI in GA response (Fridborg et al., 1999). LATERAL ROOT PRIMORDIUM1 (LRP1), another SHORT INTERNODES/STYLISH (SHI/STY) and SRS member, has been reported to interact with SHI, STY1, SRS3, SRS6 and SRS7 and affect the homeostasis and biosynthesis of auxin through the regulation of several *YUCCA* (*YUC*) genes during lateral root development (Singh et al., 2020). Meanwhile, the expression of *LRP1* is subject to feedback regulation by auxin (Singh et al., 2020). Interestingly, the crosstalk between auxin and another SRS member SRS5 has been characterized and indicates that SRS5 negatively regulates lateral root formation by repressing the expression of *LBD16* and *LBD29* (Yuan et al., 2020). Besides, SRS5 promotes photomorphogenesis activating the expression of *HY5*, *BBX21*, and *BBX22* upon exposure to light, whereas it undergoes COP1-mediated degradation *via* the 26S proteasome system in darkness (Yuan et al., 2018). In addition, SHI/STY and SRS proteins also play vital roles in floral organ development. In *Arabidopsis*, STY1 promotes stamen and gynoecium development while STY2 promotes gynoecium development (Kuusk et al., 2002; Sohlberg et al., 2006; Staldal et al., 2012).

Cotton is an important cash crop and provides raw material for textiles producing. Saline stress is a significant factor limiting crop productivity and survival (Deinlein et al., 2014; Chen et al., 2018a). In cotton, some QTLs (quantitative trait loci) and genes related to salt tolerance have been identified through either forward genetic or reverse genetic studies in recent years (Jia et al., 2016; Sun et al., 2018; Ullah et al., 2018; Dilnur et al., 2019; Li et al., 2019; Mu et al., 2019; Yasir et al., 2019; Yuan et al., 2019; Long et al., 2020). Yasir et al. identified two salt tolerance-related genes located on chromosome A10 and D10 by genome-wide association study and expression pattern analysis in upland cotton (*Gossypium hirsutum*) (Yasir et al., 2019). Dilnur et al. found two SNP loci associated with salt-stress tolerance on chromosome 7 in *G. arboretum* (Dilnur et al., 2019). Yuan et al. detected 13 QTLs using genome-wide association study and further identified 35 candidate genes responsible for cotton salt tolerance at the germination stage by RNA-seq analysis (Yuan et al., 2019). In addition, reverse genetic studies focused on cotton resistance to saline stress have made some progress in recent years. GhRaf19, a member of *MAPKKK* family in *G. hirsutum*, negatively controlled the salt tolerance by regulating the accumulation of endogenous reactive oxygen species (ROSs) in *G. hirsutum* (Jia et al., 2016). Similarly, *GhWRKY6*, a salt-induced gene, has proved to be a negative regulators of salt resistance using VIGS system (Li et al., 2019). Interestingly, another *WRKY* family member *GhWRKY6-like* had the opposite effect on cotton resistance to salt stress, which improved salt tolerance in *G. hirsutum* by activating the ABA signaling pathway and scavenging of ROSs (Ullah et al., 2018). Furthermore, protein phosphatase GhDsPTP3a interacted with a membrane protein GhANN8b and inhibited GhANN8b phosphorylation, resulting in changes of the salt induced calcium influx, the expression of GhSOS1, the outflow of sodium ions and decreased salt tolerance in *G. hirsutum* (Mu et al., 2019). In addition to regulators of cotton tolerance to salt stress, the gene structures, evolutionary relationships and expression patterns of *Na+/H+ antiporters (NHXs)* members in *G. arboreum*, *G. raimondii* and *G. hirsutum* were identified by Long et al. (Long et al., 2020). Then GhNHX1 was further proved to be located in the vacuolar system and played a crucial role in salt tolerance using VIGS system (Long et al., 2020). However, little is known about the functions of *SHI/STY* and *SRS* family genes responses to abiotic stresses in cotton.

In this study, we systematically identified 26 SRS family members in *Gossypium hirsutum* and analyze their phylogenetic relationships, protein structures, chromosomal locations, conserved motif distribution patterns, gene collinearity and expression pattern. Then, we further identified the function of a salt-inducible protein GhSRS21 under salt stress. Finally, we revealed that GhSRS21 played a negative role in salt tolerance of *Gossypium hirsutum* by controlled the balance of ROS production and scavenging. Our results will helpful to elucidate the salt response and regulation mechanism in *Gossypium hirsutum* and provide theoretical support for further in-depth research of *GhSRSs*.

## 2 Material and methods

### 2.1 Plant materials and treatment

All *G. hirsutum* plant materials used in the research were TM-1 (*Texas Marker-1*, the upland cotton genetic standard line) background. For subsequent quantitative reverse transcription (qRT)-PCR experiments, seeds of TM-1 were germinated and planted in soil under the following conditions: 12000 Lux light 16 h at 25 °C/dark 8 h at 23 °C, 80% humidity for 14 days (the first true leaf appeared). The seedlings above were divided into two groups and watered by 1/2 MS nutrient solution as the control or by 1/2 MS nutrient solution with 500 mM NaCl for 12 h.

### 2.2 Identification and property analysis of *GhSRS* genes

The genome datasets of *G. hirsutum* (ZJU, version 2.1) and *G. barbadense* (ZJU, version 1.1) were downloaded from COTTONOMICS (http://cotton.zju.edu.cn/index.htm), *G. arboreum* (WHU, version 3.0) and *G. raimondii* (NSF, version 1.0) from CottonGen (https://www.cottongen.org) and *Arabidopsis* from TAIR 10 (http://www.arabidopsis.org/). Other plant species genome datasets were downloaded from Phytozome v12.1 (https://phytozome.jgi.doe.gov/). The databases of PlantTFDB (http://planttfdb.cbi.pku.edu.cn/) and SMART (http://smart.embl.de/) were used to confirm the conserved RING-linke zinc-finger domain (DUF702). The molecular weight (MW), isoelectric point (pI) of each GhSRS were calculated using the ExPASy (https://web.expasy.org/) compute pI/Mw tool, Plant-Ploc (http://www.csbio.sjtu.edu.cn/bioinf/plant/) was for subcellular localizational prediction (Chou and Shen, 2007). All gene names and their IDs were listed in **Supplementary Table 1**.

### 2.3 Multiple alignments and phylogenetic analysis

SRS family amino acid sequences were used to perform multiple alignments by MEGA X software (Kumar et al., 2018) with MUSCLE default parameters, and then visualized using DNAMAN v7. Furthermore, the rooted and unrooted phylogenetic trees were constructed using MEGA X with Neighbor-Joining (NJ) methods, and 1000 bootstrap replicates were used to test reliability in each node and the maximum likelihood (ML) tree was constructed using “One Step Build a ML Tree” plugin in Tbtool software (Chen et al., 2020) with 5000 bootstrap replicates.

### 2.4 Gene structure, conserved motifs and synteny analysis

The gene structures of *GhSRS* genes were inferred by corresponding coding sequences. The MEME (https://meme-suite.org/meme/) program was used to identify conserved motifs in GhSRS proteins (Bailey et al., 2009). Tbtools Gene Structure View was used to draw the exon-intron structure, conserved motifs, and DUF702 domain distribution (Chen et al., 2020). The MCScanX software was used to analyze SRS protein sequences synteny and collinearity relationship between *G. hirsutum*, *G. arboreum*, and *G. raimondii*.

### 2.5 Collinearity analysis of *SRS genes* in *G. hirsutum*, *G. arboreum* and *G. raimondii*

Chromosomal positions of *GhSRS* genes were obtained from gff annotation files for *G. hirsutum* (ZJU, version 2.1). The synteny and collinearity analysis between *G. hirsutum*, *G. arboreum* and *G. raimondii* were employed by the MCScanX software (Wang et al., 2012). Tbtools Gene Location Visualize and Advanced Circos were used to draw the distribution of *GhSRS* genes chromosomal mapping and synteny relationships (Chen et al., 2020).

### 2.6 *SRS* genes expression patterns under biotic stress

To analysis the expression patterns of *SRS* genes under abiotic stress, a high-through RNA-seq datasets of leaf tissue under control and two types of stress (NaCl and PEG) treatments were obtained from COTTONOMICS (http://cotton.zju.edu.cn/) (Hu et al., 2019). Fragments per kilobase of exon per million fragments mapped (FPKM) was used for the quantification of gene expression. The clustered heatmap was drawn and normalized by the average expression levels (log2) based on FPKM values.

### 2.7 Virus-induced gene silencing assay and abiotic stress treatment

349 bp fragment of coding DNA sequence (CDS) of *GhSRS21* was amplified from *G. hirsutum* (TM-1) cDNA and constructed into *pTRV2*. Then, the recombinant vector above, *pTRV1* vector and *CLA-pTRV2* were transferred into *Agrobacterium* strain GV3101, respectively, for subsequent experiments.

Seeds of TM-1 were germinated and planted in soil under the following conditions: 12000 Lux light 16 h at 25 °C/dark 8 h at 23 °C, 80% humidity, until the cotyledons were fully opened (about 10 d after seeds germinated). The *Agrobacterium* strains containing the *GhSRS21-pTRV2* and *pTRV1* or *CLA-pTRV2* and *pTRV1* or *pTRV2* and *pTRV1* plasmids were mixed and injected into TM-1 leaves as experimental groups, positive and negative controls, respectively. Cotton infected by *Agrobacterium tumefaciens* was cultured under the conditions above until the white striped leaf or albino phenotype appeared in the positive control (about 10 d after infected). Half of the experimental and negative control groups was watered by 1/2 MS nutrient solution as the control while the other half was watered by 500 mM NaCl regularly after every 3 days until the phenotypes appeared (about 14 d after treatment).

### 2.8 RNA isolation and qRT-PCR

Total RNA was isolated from leaf tissues of TM-1 under salt stress treatment at 0 h, 1 h, 3 h, 6 h and 12 h using RNAprep pure Plant Kit (code: DP432, Tiangen, Beijing, China). First-strand cDNA was synthesized using Hifair 1st Strand cDNA Synthesis SuperMix for qPCR (YEASEN Biotech, Shanghai, China). The qRT-PCR was performed using Hieff qPCR SYBR GreenMaster Mix (YEASEN Biotech, Shanghai, China). The cotton ubiquitin gene *UBQ7* was used as the internal control for the relative expression calculation. RNA isolation and qRT-PCR was manipulated based on the manufacturer’s instructions. The primers used for qRT-PCR were listed in **Supplementary Table 2**.

### 2.9 Measurement of chlorophyll content and determination of malondialdehyde, H_2_O_2_, O^2·-^ and antioxidant enzymes activities

The samples of cotton leaves obtained from negative control groups and *GhSRS21* VIGS lines (experimental groups) treated with or without salt and PEG6000 in method 2.6 were used for measurement of chlorophyll content and determination of malondialdehyde, H_2_O_2_, O^2·-^ and antioxidant enzymes activities. The detail method for determination of malondialdehyde, H_2_O_2_, O^2·-^ and antioxidant enzymes activities was performed as described by Zhan et al. (Zhan et al., 2021), and method for measurement of chlorophyll contents were performed according to our previously described methods (Liu et al., 2019).

### 2.10 Subcellular localization

The CDS of GhSRS21 fused with GFP (green fluorescent protein) tag at the 3′-end was ligated into the *pCAMBIA1300* vector. The recombinant plasmid above was mixed with the nuclear marker *NLS-mCherry* and co-transformed into *Arabidopsis* mesophyll protoplasts as described (Yoo et al., 2007). The protoplasts were observed and photographed by a fluorescence microscopy (Zeiss Imager.A2, Germany). The primers used above were listed in **Supplementary Table 2**.

### 2.11 RNA-seq and KEGG analysis

Total RNA was isolated from leaf tissues of negative control groups and *GhSRS21* VIGS lines when the albino-like appearance on the leaves of positive control was observed. The fragments were purified by agarose gel electrophoresis and sequenced with NovaSeq 6000 Sequencer (Illumina Inc., San Diego, CA, USA) with a read length of 150 bp. Three biological replicates were performed separately. The raw data were filtered with fastp (https://github.com/OpenGene/fastp) (Chen et al., 2018b). The reads filtered above were then mapped to the cotton reference genome using HISAT2 software (http://ccb.jhu.edu/software/hisat2) (Kim et al., 2015; Zhang et al., 2015). Transcript analysis was performed using StringTie (https://ccb.jhu.edu/software/stringtie/) (Pertea et al., 2015), and differential expression genes (DEGs) analysis was performed by DEseq2 (Love et al., 2014), FPKM > 1.0 (FPKM, Fragments Per Kilobase of exon per Million mapped reads) were regarded as valid DEGs. Subsequently, KEGG annotations were performed using the online software EggNOG-Mapper (http://eggnog-mapper.embl.de/) and KEGG enrichment analysis were performed using Tbtools software (Chen et al., 2020).

### 2.12 Data processing and analysis

Statistical analysis was performed using SPSS version 23.0 statistical software (SPSS, Inc., Chicago, IL, USA). All data were subjected to analysis of variance (One-way ANOVA) and mean comparisons were carried out by Ducan’s multiple range test (p < 0.05).

## 3 Results

### 3.1 Identification of *SRS* genes in three cotton species

To identify all members of the *SRS* gene family in cotton, the conserved RING-like zinc-finger domain (DUF702) (Pfam ID: PF05142) from the Pfam databases (http://pfam.xfam.org/) were employed as queries to search against three main representative cotton species. Then, the puatative protein sequences using the PlantTFDB (http://planttfdb.cbi.pku.edu.cn/) and SMART (http://smart.embl.de/) databases to confirm the predicted functional domains contained DUF702 (PF05142) families. A total of 53 SRS members were identified in *G*. *arboreum*, *G*. *raimondii*, and *G*. *hirsutum*, of which 14 were *GaSRS* genes, 13 were *GrSRS* genes, and 26 were *GhSRS* genes (13 *SRS*s from At subgenome, 13 *SRS*s from Dt subgenome). Therefore, we named them GhSRS1~GhSRS26 based on their gene ID number and genomic distribution. The encoded protein length of *GhSRS* genes ranging from 195 (GhSRS3) aa to 434 (GhSRS22) aa, and molecular weight (MW) from 22116.24 Da to 45074.19 Da, isoelectric point (pI) varying from 5.49 (GhSRS25) to 9.22 (GhSRS26), in addition to predicting the subcellular location of all GhSRS members, 16 of GhSRS proteins were nucleus-localized. Other basic information for all SRS members in three cotton species were listed in **Supplementary Table 3**.

**Figure 1:**
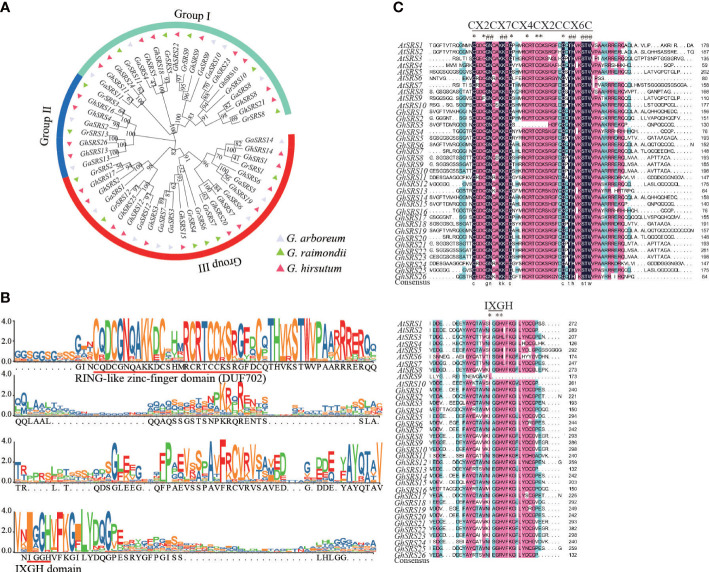
Phylogenetic and domain structure analysis of the *SRS* family proteins in three cotton species. **(A)** the unrooted phylogenetic tree of the *SRS* family in *G arboreum*, *G raimondii*, and *G hirsutum* at the amino acid sequences level using Neighbor-Joining method in MEGA X with 1000 bootstrap. The subgroup was divided according to the value on the node at the root of the evolutionary tree (the value>=80: all genes under this node belong to one subgroup; the value<80: all genes under this node can be further divided into two or more subgroups). **(B)** The conserved RING-like zinc-finger domain (CX_2_CX_7_CX_4_CX_2_C_2_X_6_C). The alignment domain sequence was obtained from CottonGen (https://www.cottongen.org/). **(C)** Alignment of conserved RING-like zinc-finger domain and the IXGH domain in *A thaliana* and *G hirsutum*.

To better understand the phylogenetic and evolution relationships of *SRS* genes in cotton, the unrooted phylogenetic tree constructed by MEGA X revealed the *SRS* family genes can be divided into three subgroups (**Figure 1A**). The number of *SRS* genes in *G*. *hirsutum* was almost the sum of the number of those in *G*. *arboreum* and *G*. *raimondii*, which was consistent with polyploidy and whole-genome duplication (WGD) events during hybridization.

We found that almost all members of this family contained a RING-like zinc-finger domain (CX_2_CX_7_CX_4_CX_2_C_2_X_6_C) through sequence alignment of amino acid residues. But lacking part of the RING domain in GhSRS3 may leading to a decrease in the binding ability to DNA, RNA, protein, and lipid substrates (**Figure 1B**). Moreover, GhSRSs also share a IXGH domain except GhSRS13/26, and this conserved region longer than IXGH domain in GmSRSs (**Figure 1C**) (Zhao et al., 2020).

### 3.2 Phylogenetic analysis of *SRS* genes

**Figure 2:**
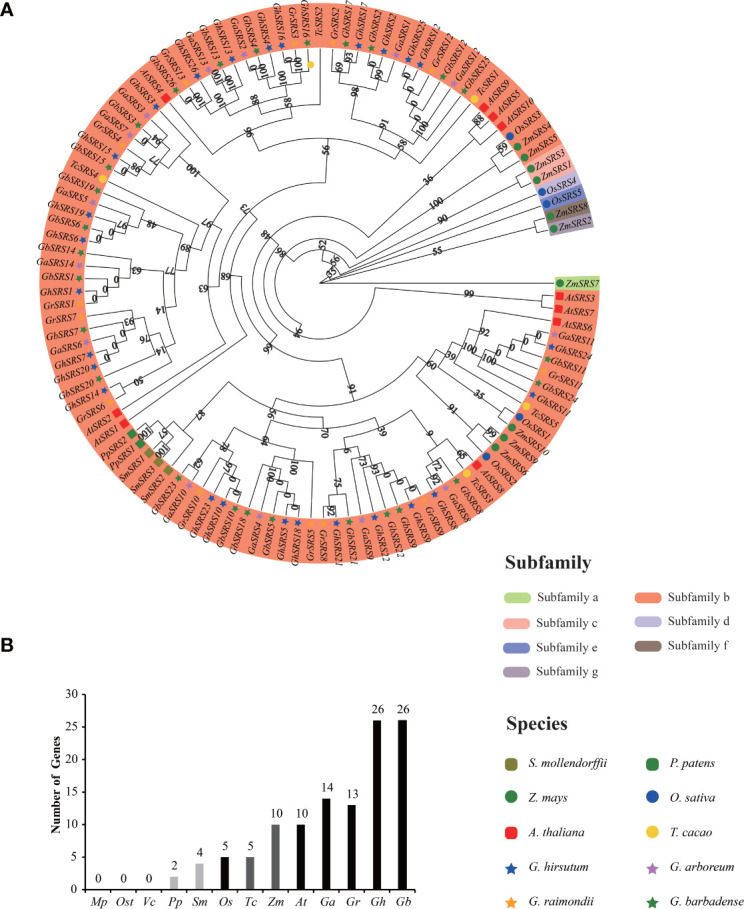
Phylogenetic and evolution relationships of the *SRS* gene family among different organisms **(A)** the rooted phylogenetic tree of the *SRS* gene family at the amino acid sequences level using the Maximum Likelihood method in Tbtools with 5000 bootstrap. The subgroup was divided according to the value on the node at the root of the evolutionary tree (the value>=80: all genes under this node belong to one subgroup; the value<80: all genes under this node can be further divided into two or more subgroups). **(B)** Comparisons of *SRS* gene numbers across a wide range of organisms. The prefix *Mp*, *Ost*, *Vc*, *Pp*, *Sm*, *Os*, *Tc*, *Zm*, *At*, *Ga*, *Gr*, *Gh* were used to describe the names of *M. pusilla*, *Os. tauri*, *V. carteri*, *P. patens*, *S. moellemdorffii*, *O. sativa*, *T. cacao*, *Z. mays*, *A thaliana*, *G arboreum*, *G raimondii*, *G barbadense*, *G hirsutum*, respectively.

To investigate the evolutionary relationships of *SRS* gene family, we constructed a separate rooted phylogenetic tree using 13 plant species genome datasets from lower aquatic to higher terrestrial plants. We totally identified 115 genes in different moss (2 in *P*. *patens*), fern (4 in *S*. *moellendorffii*), monocotyledons (5 in *O*. *sativa*, 10 in *Z*. *mays*), and dicotyledons (5 in *T*. *cacao*, 10 in *A*. *thaliana*, 14 in *G*. *arboreum*, 13 in *G*. *raimondii*, 26 in *G. barbadense* and 26 in *G*. *hirsutum*), while no *SRS* gene was found in picophytoplankton (*M*. *pusilla*) and algae (*Os*. *tauri*, *V*. *carteri*) (**Figure 2A, B**). Results revealed that *SRS* gene first appeared in moss (P. patens), and the number of *SRS* gene increased dramatically in *G. barbadense* and *G*. *hirsutum* (**Figure 2B**).

The *SRS* genes in multiple plant species can be divided into 7 clades and named a to g subfamilies. The *SRS* subfamiliy a and c-gonly existed in monocotyledon and each subfamiliy above contained only one or two members. However, SRS subfamily b was the largest subfamily and contained 108 *SRS* members including all *SRS* genes derived from the above dicotyledons and some SRS genes derived from the above monocotyledons (**Figure 2A**). Furthermore, the number of *GhSRS* genes in *G. barbadense* or *G. hirsutum* were almost the sum of *SRS* genes in *G*. *arboreum* and *G*. *raimondii* (**Figure 2B**), which confirmed the ideas that allotretraploid cotton (*G. barbadense* and *G*. *hirsutum*) evolved from hybridization and polyploidization between two diploid cotton species (*G*. *arboreum* and *G*. *raimondii*).

### 3.3 Gene structure and domain analysis

**Figure 3:**
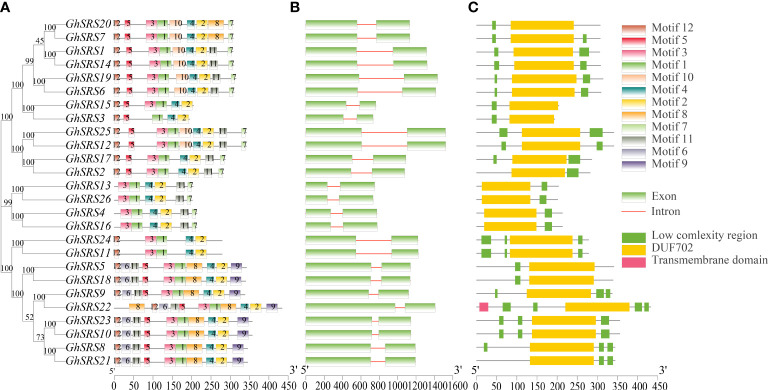
Conserved motifs, gene structures and conserved domains of SRS family members **(A)** Conserved motifs identification through Multiple EM for Motif Elicitation (MEME) **(B)** Gene structures of SRS proteins, green rectangle represents the exon of SRS genes and line represents intron **(C)** Conserved motifs of SRS proteins, the blue rectangle represents the SRS conserved domain containing the RING-like zinc-finger domain (CX_2_CX_7_CX_4_CX_2_C_2_X_6_C) and named DUF702.

To figure out the structure similarity of the *SRS* family in cotton, the full-length protein sequence of 26 GhSRSs were aligned to display phylogenetic tree with conserved motifs, exon-intron, and domain structure. Conserved motifs in the SRS protein sequences were performed by MEME online service (https://meme-suite.org/meme/), 12 different motifs were identified and distributed on the GhSRS protein sequences. GhSRSs in the same cluster shared similar conserved motif composition, especially all GhSRS members contained some conserved motifs (motif 1, motif 2, and motif 4) (**Figure 3A**). And the motif number of each protein ranging from five to eleven (**Figure 3A**). Then, we performed the exon-intron gene structure analysis by comparing genomic sequence to the extended cDNA sequence (CDS) of *GhSRSs*. The number of coding exons of *GhSRSs* in *Gossypium hirsutum* was conserved, as they all contained two exons (**Figure 3B**). However, the length of the introns of the *GhSRS* genes was largely variable, ranging from 73 bp to 497 bp (**Figure 3B**). The domain structure of SRS proteins was analyzed using the SMART protein-domain search interface (http://smart.embl.de/), and the results showed all GhSRSs shared a conserve domain named DUF702, containing the RING-like zinc-finger domain (CX_2_CX_7_CX_4_CX_2_C_2_X_6_C) (**Figure 3C**). Interestingly, only the GhSRS22 possessed a transmembrane domain. The results above indicated that the GhSRS22 was likely to be a membrane-bound protein, which was consistent with the result listed in **Supplementary Table 3**.

### 3.4 Genomic distribution and synteny analysis

**Figure 4:**
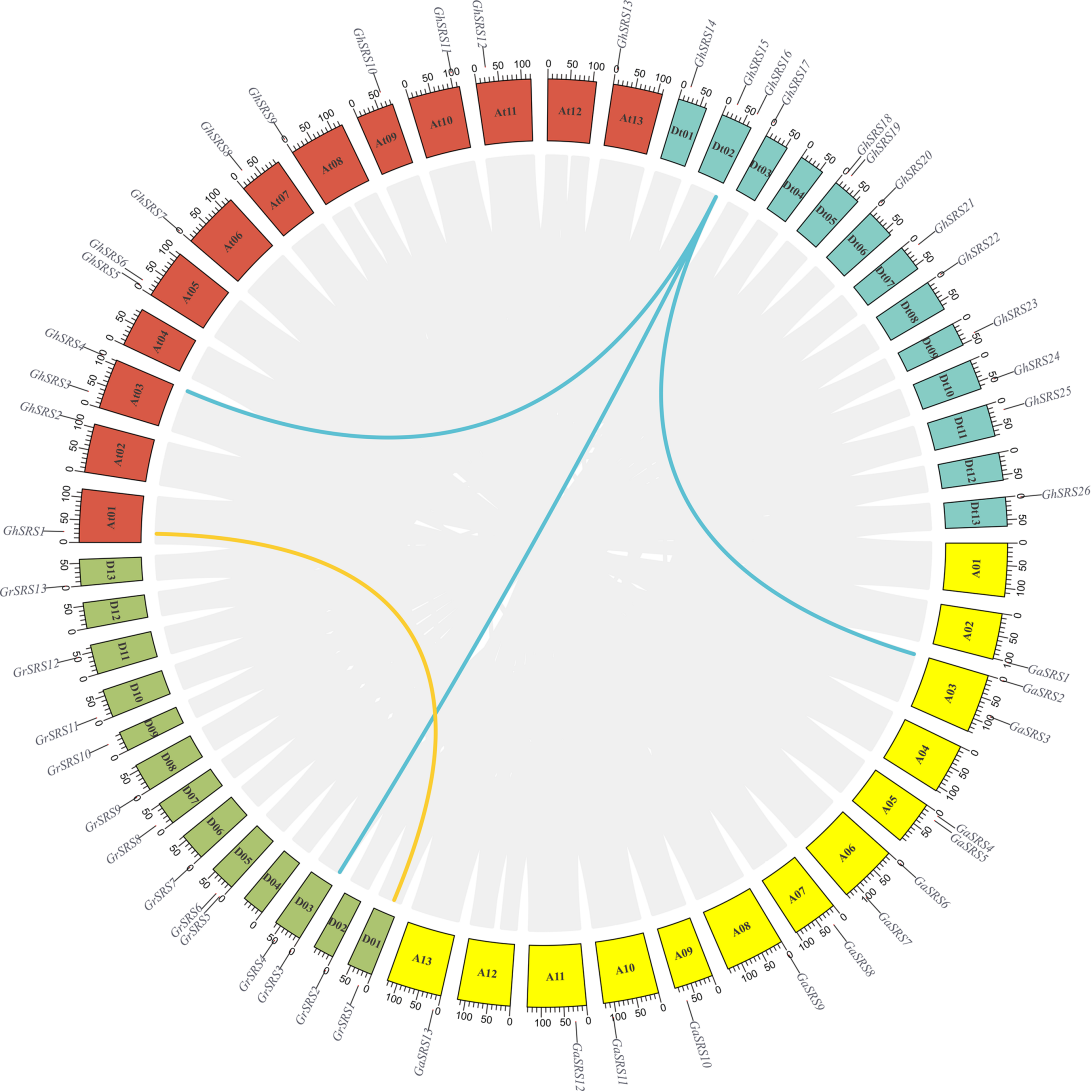
Collinearity analysis of *SRS* genes between *G. hirsutum*, *G. arboreum* and *G. raimondii*. A01 – A13 represent 13 chromosomes in *G. arboreum*, D01 – D13 represent 13 chromosomes in *G. raimondii*, At01 – At13, Dt01 – Dt13 represent 13 At subgenome chromosomes and 13 Dt subgenome chromosomes in *G. hirsutum*. Gene joined by yellow and blue lines are the product of gene duplication.

To investigate the chromosomal distribution and the duplication events of the *SRS* family in cotton, chromosomal distribution and collinearity analysis was performed. In *G. arboreum*, 13 *SRS* family members were distributed on chromosomes At02, At03, At05, At06, At07, At08, At09, At010, At11, At13. Meanwhile, a total of 13 *SRS*s were unevenly distributed on chromosomes Dt01, Dt02, Dt03, Dt05, Dt06, Dt07, Dt08, Dt09, Dt10, Dt11, Dt13 (**Figure 4**). Interestingly, although the number of *SRS* genes in *G. hirsutum* were not altered, their distributions on chromosomes in *G. hirsutum* displayed differences compared with those in *G. arboreum* and *G. raimondii*, implying structural variation on chromosomes during the process of cotton polyploidization. For instance, *GrSRS1* was located on chromosome Dt01 in *G. raimondii* while there was no *SRS* gene on chromosome At01 in *G. arboreum* (**Figure 4**). However, in *G. hirsutum*, *GhSRS1* was located in chromosome At01 and exhibited high gene collinearity with *GrSRS1*, probably resulting from the gene duplication and interchromosomal translocation. In addition, one *SRS* gene loss occurred in chromosome Dt03 (**Figure 4**). In summary, the *SRS* genes in *G. hirsutum* were unevenly distributed on all chromosomes except for the chromosomes At04, At12, Dt04, Dt12. Besides, *SRS* gene duplication and loss events occurred during ancestral allopolyploidization of *G. hirsutum*.

### 3.5 Expression patterns of *GhSRS*s under salt and drought stresses

**Figure 5:**
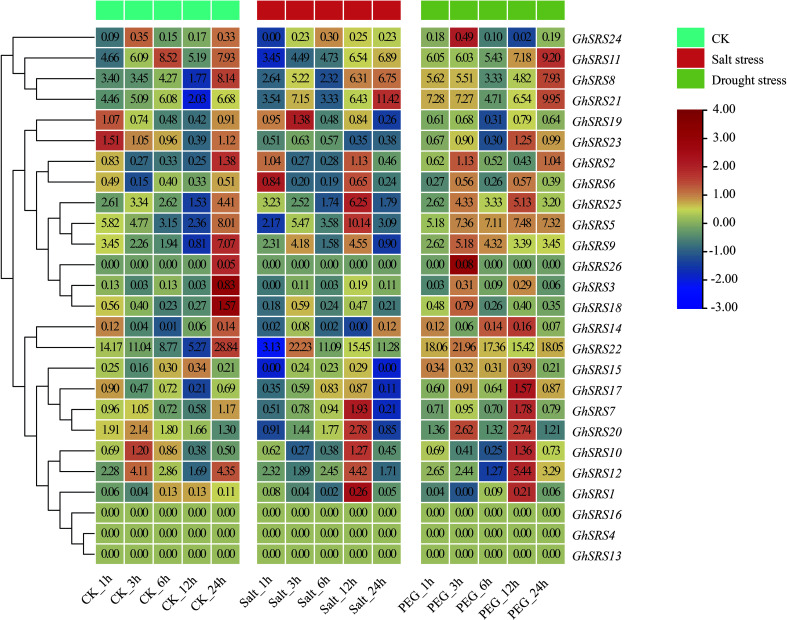
Expression patterns of *GhSRS* family members under salt and drought stress. Color intensity displayed in the heatmap are the Log_2_ transformed RPKM gene expression value. The origin FPKM values were showed in the squares. CK represents the control group.

To better understand the function of *GhSRS* genes under salt and drought stresses in *Gossypium hirsutum*, the expression pattern of *GhSRSs* in response to salt and drought stress was examined using the FPKM values of *GhSRSs* extracted from COTTONOMICS (http://cotton.zju.edu.cn/index.htm) (**Figure 5**). The results showed that most *GhSRSs*, such as *GhSRS1*, *GhSRS2*, *GhSRS3*, *GhSRS4*, *GhSRS6*, *GhSRS7*, *GhSRS10*, *GhSRS12*, *GhSRS13*, *GhSRS14*, *GhSRS15*, *GhSRS16*, *GhSRS17*, *GhSRS18*, *GhSRS19*, *GhSRS20*, *GhSRS23*, *GhSRS24*, *GhSRS25*, *GhSRS26*, expressed at a very low level (average FPKM<5) under control, salt and drought stress (**Figure 5**). Of the remaining 6 genes, *GhSRS21* was induced by salt stress (3h, 12h and 24h after salt treatment) while the transcripts of *GhSRS5, GhSRS22* showed high level accumulation after drought treatment (**Figure 5**), indicating their potential roles in salt or drought tolerance.

### 3.6 GhSRS21 subcellular localization

**Figure 6:**
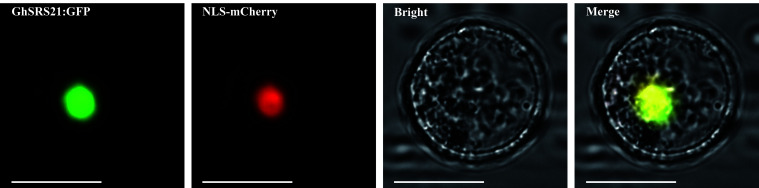
Subcellular location of GhSRS21. NLS-mCherry: nuclear localization sequence fused with a reporter gene mCherry, which is used as nuclear localization marker. Bar=10 μm.

To further confirm the nuclear localization of GhSRS21 and presume its potential role in regulation of the expression of eukaryotic genes, a nuclear localization sequence fused with the mCherry (NLS- mCherry) was used as nuclear localization marker and cotransformed with the plasmid (GhSRS21 ORF fused with GFP) to the *Arabidopsis* protoplasts. There was overlapping between green fluorescence and red fluorescence, indicating GhSRS21 located in the nucleus (**Figure 6**).

### 3.7 GhSRS21 negatively regulates salt tolerance in a manner dependent on reactive oxygen species metabolic process in *G. hirsutum*

**Figure 7:**
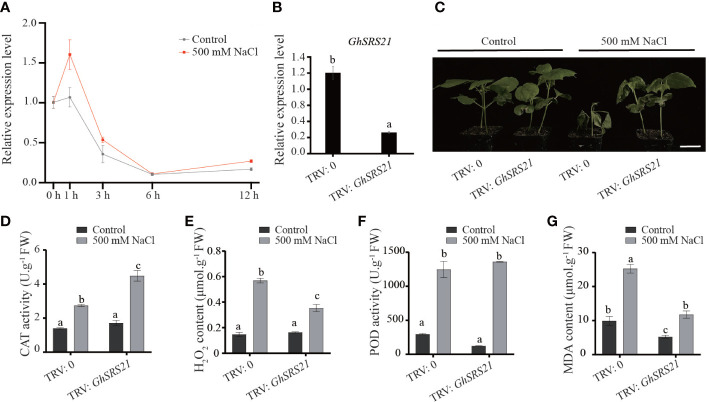
Phenotypic identification of *GhSRS21* VIGS line under salt stress. **(A)** The expression levels of *GhSRS21* in the negative control and *GhSRS21* VIGS lines under control and 500 mM NaCl at 0 h, 1 h, 3 h, 6 h, 12 h. **(B)** The expression levels of *GhSRS21* in the negative control and *GhSRS21* VIGS lines. Each qPCR reaction was performed with three technical replicates. **(C)** Phenotypic identification of *GhSRS21* VIGS line under control and 500 mM NaCl treatment. TRV2: 0 and TRV2: *GhSRS21* represent the negative control and *GhSRS21* VIGS line, respectively. Bar = 4 cm **(D–G)**SOD activity, POD activity and H_2_O_2_, MDA content in the negative control and *GhSRS21* VIGS lines under control and 500 mM NaCl treatment. FW indicates fresh weight. Significant differences are determined using one-way ANOVA and Ducan’s Multiple Range Test, as indicated with different letters at P < 0.05 significance level.

The expression pattern of *GhSRS21* under salt stress has been described previously using the COTTONOMICS public database ((http://cotton.zju.edu.cn/index.htm)) (**Figure 5**). To further confirm the expression pattern of *GhSRS21* in response to salt stress, RT-qPCR was performed and the results revealed that the *GhSRS21* gene expression was highly induced by salt stress (**Figure 7A**). Subsequently, the function of *GhSRS21* under salt stress conditions was determined using the VIGS (virus-induced gene silencing) system. When the albino-like appearance on the leaves of positive control was observed (**Supplementary Figure 1**), we examined the expression of *GhSRS21* in negative control and *GhSRS21* VIGS lines. The data showed that the expression levels of *GhSRS21* were significantly decreased in *GhSRS21* VIGS lines compared with those in negative control (**Figure 7B**). To further understand the role of *GhSRS21* in *G. hirsutum* under salt stress, salt treatment was performed and silenced *GhSRS21* in *TM-1* resulted in enhanced salt tolerance (**Figure 7C**). In addition, we further detected the activities of antioxidant enzymes and relevant physiological indicators of negative control and VIGS plant under control and salt treatment. The results exhibited the increased activity of catalase (CAT), peroxidase (POD) and increased content of hydrogen peroxide (H_2_O_2_), malondialdehyde (MDA) significantly in both *GhSRS21* VIGS cottons and negative control after salt treatment (**Figures 7D–G**). However, a significantly higher CAT activity was observed in the *GhSRS21* VIGS lines compared with that of the negative control after salt treatment (**Figure 7D**). Meanwhile, the content of H_2_O_2_ and MDA in *GhSRS21* VIGS lines was significantly lower than that in the negative control after salt treatment (**Figures 7E, G**). Taken together, GhSRS21 negatively regulates salt tolerance in *G. hirsutum* through increased antioxidant capacity of cotton.

**Figure 8:**
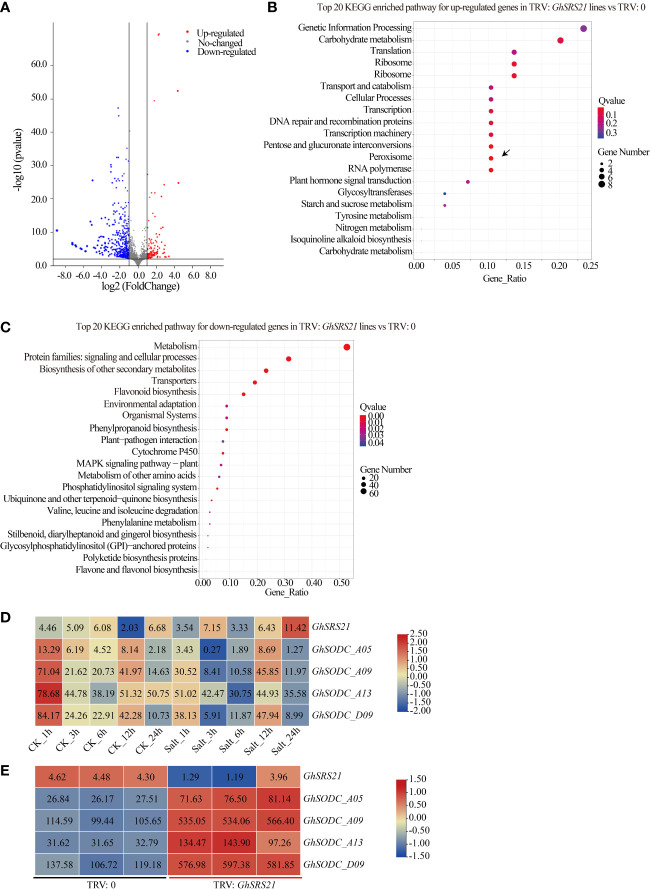
Transcriptome analysis of *GhSRS21* VIGS lines.**(A)** Volcano map of DEGs (*GhSRS21* VIGS lines *vs* negative control). **(B, C)** KEGG pathway analysis for up-regulated genes and down-regulated genes in *GhSRS21* VIGS lines *vs* negative control (TRV: *GhSRS21* lines represent the *GhSRS21* VIGS lines and TRV: 0 represent the negative control), respectively. **(D)** Expression pattern of *GhSRS21* and SODCs under control and salt treatment. **(E)** Expression pattern of *GhSRS21* and SODCs in *GhSRS21* VIGS lines *vs* negative control. Heatmap in **(D, E)** was normalized by the average log_2_ of FPKM values and the numbers in rectangle indicate FPKM values.

To explore the potential mechanism of GhSRS21 in regulation of H_2_O_2_ production, genes differentially expressed in the negative control and *GhSRS21* silenced plants were analyzed *via* the transcriptome data. The results indicated that the number of down-regulated genes (306) was more than that of up-regulated genes (71) in *GhSRS21* silenced lines compared with the negative control (**Figure 8A**). Furthermore, the enrichment of the differentially expressed genes (DEGs) in the KEGG pathway was analyzed. Most up-regulated genes were enriched in the pathways of genetic information processing, carbohydrate metabolism, transcription, translation, peroxisome, etc, while the down-regulated genes showed significant enrichment on metabolism, transporters, flavonoid biosynthesis, etc (**Figures 8B, C**). There were four up-regulated genes (*GH_A05G0875*, *GH_A09G1066*, *GH_A13G1914*, *GH_D09G1018*), enriched in peroxisome KEGG pathway, belong to *copper/zinc superoxide dismutase* (*SODC*), and shared potential biological functions in ROS (reactive oxygen species) scavenging. To further figure out their roles in salt resistance of the upland cotton, the expression pattern of the mentioned *SODCs* above were analyzed using the public COTTONOMICS Database. The results showed that the four *SODCs* above were significantly suppressed by salt stress while *GhSRS21* exhibited the opposite expression pattern under salt treatment (**Figure 8D**). Moreover, the four *SODCs* were induced when the *GhSRS21* was repressed in the VIGS lines (**Figure 8E**). In summary, the *GhSRS21* negatively regulates salt tolerance in a manner dependent on reactive oxygen species metabolic process and probably by negative regulation of *SODCs* expression.

## 4 Discussion

The global soil salinization is major and growing ecological problems due to the rising sea level from global climate warming and inappropriate irrigation practice (Munns and Gilliham, 2015; Zhou et al., 2017). Salinity stress is one of the most important constraints on crop yield (Deinlein et al., 2014). Salt stress includes three types: osmotic stress, ionic stress, and oxidative damage (Ismail and Horie, 2017; Yang and Guo, 2018). Plants produce compatible osmolytes such as proline and soluble sugars and increase the cellular osmolarity to maintain the capability to absorb water under salt stress, which help the plants under stress in osmotic adjustment (Deinlein et al., 2014; Park et al., 2016; Yang and Guo, 2018). On the other hand, Plants exposure to salt stress induces overproduction of reactive oxygen species (ROS), which results in membrane injury and increased MDA production (Choudhury et al., 2017). The antioxidant enzymatic system, such as superoxide dismutase (SOD), ascorbate peroxidase (APX), CAT, guaiacolperoxidase (GPX) and POD, is responsible for scavenging of ROS induced by salt stress (Yang and Guo, 2018).

The SHI/STY and SRS members are plant-specific transcription factors (Zhao et al., 2020). Available researches of SRS transcription factors have focused on their role in regulation of plant growth and development (Zhao et al., 2020). However, the role of *SRS* transcription factors participated in plant’s resistance to abiotic stress is few reported. Besides, the function of SRS members in *Gossypium hirsutum* is largely unclear. In this study, we identified 26 *GhSRS*s in *G. hirsutum* and most of them were predicted to located in the nucleus (**Supplementary Table 3**), indicating their crucial roles in regulating nuclear gene expression. Then the conserved domain of the GhSRSs was analyzed and the result was consistent with the reported literatures (Fridborg et al., 2001; Zhao et al., 2020). Most *SRS* members in *Gossypium hirsutum* share a RING-like zinc-finger domain (CX_2_CX_7_CX_4_CX_2_C_2_X_6_C) and a IXGH domain (**Figures 1B, C**), which is responsible for biological macromolecules binding (DNA, RNA, protein and/or lipid substrates) (Klug, 1999) and transcriptional activation (Fridborg et al., 2001; Singh et al., 2020), respectively. Nevertheless, defective in RING-like zinc-finger domain of GhSRS3 and defective in IXGH domain of GhSRS13/26 most likely result in loss of function (**Figures 1B, C**). Furthermore, we identified the *SRS* genes in *M*. *pusilla*, *Os*. *tauri*, *V*. *carteri*, *P*. *patens*, *S*. *moellemdorffii*, *O*. *sativa*, *T*. *cacao*, *Z*. *mays*, *A*. *thaliana*, *G*. *arboreum*, *G*. *raimondii*, *G. barbadense*, *G*. *hirsutum* and investigated their evolutionary relationships. Interestingly, the *SRS* genes were missing in some algae (*M. pusilla*, *Os. Tauri* and *V. carteri*) but appeared in the land plants (*P*. *patens*, *S*. *moellemdorffii*, *O*. *sativa*, *T*. *cacao*, *Z*. *mays*, *A. thaliana*, *G*. *arboreum*, *G*. *raimondii*, *G. barbadense* and *G*. *hirsutum*) (**Figures 2A, B**). The results above indicated the potential role of *SRS* genes in the transition of plant from the water to the land. Besides, all members in SRS subfamily a and c-g were derived from monocots (*O. sativa* and *Z. mays*) (**Figure 2A**), indicating that these genes might evolve separately and played a specific role in monocots grown and stress defense. Our result above is also in agreement with the work of Yang et al. (Yang et al., 2020). Besides, Tetraploid cotton (represented by *G. barbadense* and *G. hirsutum*; AD1) originated from an allopolyploidization event between an A-genome (*G. herbaceum*- or G. arboreum-like) and a D-genome (G. raimondii-like) diploid species circa 1 to 2 million years ago (Wendel and Grover, 2015; Hu et al., 2019). The number of *GhSRS* genes in *G. barbadense* or *G. hirsutum* were almost the sum of *SRS* genes in *G*. *arboreum* and *G*. *raimondii* (**Figure 2B**), which confirmed the ideas that allotretraploid cotton (*G. barbadense* and *G*. *hirsutum*) evolved from hybridization and polyploidization between two diploid cotton species (*G*. *arboreum* and *G*. *raimondii*). Subsequently, we analyzed the gene structures and protein domains of *GhSRSs*. The gene structures are well conserved in cotton genome (all contained only one intron) (**Figure 3B**) in contrast to those in soybean, maize and alfalfa (He et al., 2020; Zhao et al., 2020; Yang et al., 2021). Besides, GhSRS22 is a unique member of the GhSRSs, containing the transmembrane domain (**Figure 3C**) and belonging to plant membrane-bound transcription factors. The plant membrane-bound transcription factors are usually located in cellular membranes and represented in inactive state (Liu et al., 2018; De Backer et al., 2022). However, the plant membrane-bound transcription factors are activated and relocate to the nucleus by protease cleavage of themselves in response to an intra- or extra-cellular trigger (Kim et al., 2010; De Backer et al., 2022). Consequently, GhSRS22 could play a crucial role in the regulation of the gene expression process under specific conditions.

To further explore biological functions of *GhSRSs* under multiple abiotic stresses, we analyzed their expression patterns in response to salt and drought stress in *G. hirsutum* and *GhSRS21*, a salt-inducible gene, was identified (**Figure 5**). The results of subcellular localization showed GhSRS21 belonged to the nuclear transcription factors (**Figure 6**), which was consistent with our predictions (**Supplementary Table 3**). The results above also implied its biological functions involved in regulation of nuclear gene expression. Furthermore, *GhSRS21* silenced in *Gossypium hirsutum* L. increased cotton resistance to salt (**Figures 7A–C**), further conforming the negative regulatory role of *GhSRS21* in cotton tolerance to salt. Similarly, Zhao et al. demonstrated that *GmSRS18* negatively controlled drought and salt resistance in transgenic *Arabidopsis* (Zhao et al., 2020). Therefore, the SRSs tend to be negative regulators when plants are subjected to various abiotic stresses. H_2_O_2_, MDA and ROS are important markers reflecting the severity of the salinity stress while CAT and POD are responsible for ROS and H_2_O_2_ scavenging (Wang et al., 2013; Yang and Guo, 2018). Our further researches indicated that GhSRS21 modulated salt stress tolerance of cotton through negative regulation of CAT activity and H_2_O_2_ scavenging (**Figures 7E, F**). Nevertheless, the mechanism by which GhSRS21 negatively regulated H_2_O_2_ scavenging is unclear. Thus, the transcriptome sequencing was performed using the negative control and *GhSRS21* VIGS lines and the results reveal that the up-regulated DEGs (*GhSRS21* VIGS lines compared with negative control) were enriched in peroxisome pathway, containing four copper/zinc superoxide dismutase (SODC) genes (**Figure 8B**). Meanwhile, the four SODC genes above and *GhSRS21* showed opposite expression pattern when cotton were subjected to salt stress (**Figure 8D**). SODC, responsible for ROS scavenging, play a crucial role in plant salt tolerance (Sunkar et al., 2006). It follows that GhSRS21 controlled salt sensitivity of cotton by regulation of the balance between ROS production and scavenging. In general, we identified a negative regulatory transcription factor GhSRS21 involving in cotton salt tolerance may provide valuable candidates for efforts toward the genetic improvement of cotton.

## Data availability statement

The datasets presented in this study can be found in online repositories. The names of the repository/repositories and accession number(s) can be found below: https://www.ncbi.nlm.nih.gov/, PRJNA893181.

## Author contributions

CS conceived and designed the research. CS, LY, and SZ performed the experiments. CS, LY, and QG analyzed the data. CS, QG and MW contributed to writing the manuscript. CS, LY, QG and MW modified and revised the manuscript. All authors contributed to the article and approved the submitted version.
