# Supplementary material for: Genome-wide characterization of the SHORT INTER-NODES/STYLISH and Shi-Related Sequence family in Gossypium hirsutum and functional identification of GhSRS21 under salt stress
Source: Front Plant Sci. 2023 Jan 4;13:1078083. doi: 10.3389/fpls.2022.1078083 (PMC9846857; doi:10.3389/fpls.2022.1078083)
Supplement: Supplementary file 1 [file DataSheet_1.pdf]

Supplementary Material

1.1 Supplementary Figure

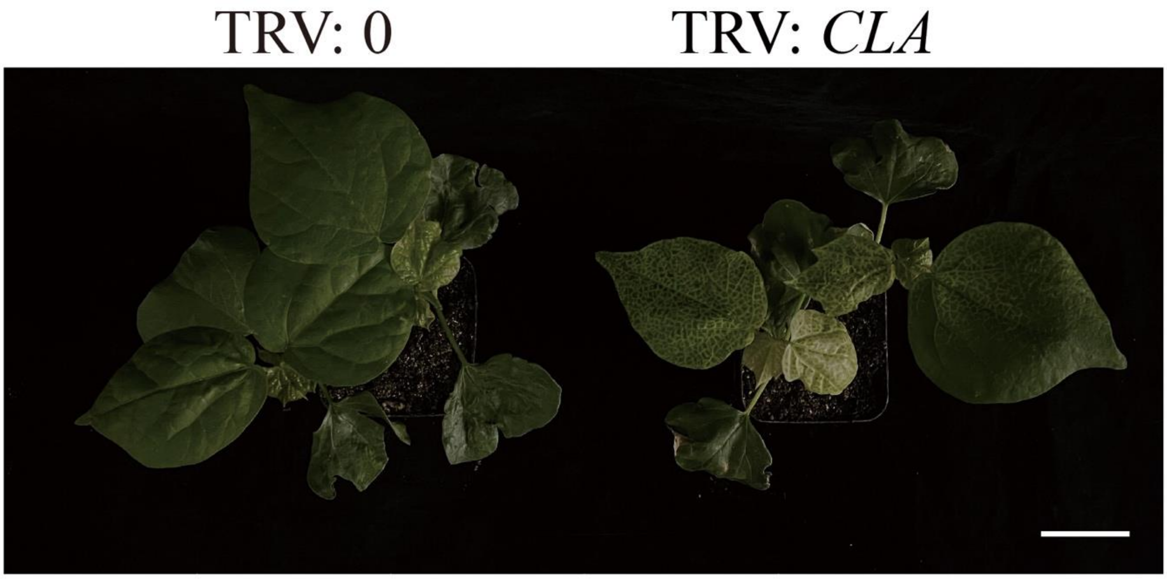

Supplementary Figure 1. The albino phenotype after *GhCLA* silencing by VIGS. The TRV: 0, TRV: *CLA* represent the negative control and positive control, respectively. Bar = 4cm

1.2 Supplementary Table

Supplementary Table 1. *SRS* gene names and IDs in *M. pusilla, Os. tauri, V. carteri, P. patens, S. moellemdorffii, O. sativa, T. cacao, Z. mays, A. thalianathailand, G. arboreum, G. raimondii, G. barbadense* and *G. hirsutum*

Gene name	Gene ID
GrSRS1	D5.v1.pred_00023068-RA
GrSRS2	D5.v1.pred_00038731-RA
GrSRS3	D5.v1.pred_00024875-RA
GrSRS4	D5.v1.pred_00026495-RA
GrSRS5	D5.v1.pred_00000811-RA
GrSRS6	D5.v1.pred_00002050-RA
GrSRS7	D5.v1.pred_00030577-RA
GrSRS8	D5.v1.pred_00034781-RA
GrSRS9	D5.v1.pred_00008943-RA
GrSRS10	D5.v1.pred_00029008-RA
GrSRS11	D5.v1.pred_00013020-RA
GrSRS12	D5.v1.pred_00006884-RA
GrSRS13	D5.v1.pred_00018967-RA
GbSRS1	GB_A01G1119
GbSRS2	GB_A02G1975
GbSRS3	GB_A03G0950
GbSRS4	GB_A03G2307
GbSRS5	GB_A05G0734
GbSRS6	GB_A05G1915
GbSRS7	GB_A06G0061
GbSRS8	GB_A07G1302
GbSRS9	GB_A08G0133
GbSRS10	GB_A09G1366
GbSRS11	GB_A10G2155
GbSRS12	GB_A11G1884
GbSRS13	GB_A13G0309
GbSRS14	GB_D01G1191
GbSRS15	GB_D02G1045
GbSRS16	GB_D02G2451
GbSRS17	GB_D03G0110
GbSRS18	GB_D05G0721
GbSRS19	GB_D05G1941
GbSRS20	GB_D06G0062
GbSRS21	GB_D07G1300
GbSRS22	GB_D08G0133
GbSRS23	GB_D09G1213
GbSRS24	GB_D10G2144
GbSRS25	GB_D11G1930
GbSRS26	GB_D13G0305
GhSRS1	GH_A01G1110
GhSRS2	GH_A02G1950
GhSRS3	GH_A03G0939
GhSRS4	GH_A03G2224
GhSRS5	GH_A05G0731
GhSRS6	GH_A05G1887

GhSRS7	GH_A06G0057
GhSRS8	GH_A07G1312
GhSRS9	GH_A08G0136
GhSRS10	GH_A09G1252
GhSRS11	GH_A10G2026
GhSRS12	GH_A11G1874
GhSRS13	GH_A13G0304
GhSRS14	GH_D01G1151
GhSRS15	GH_D02G0996
GhSRS16	GH_D02G2394
GhSRS17	GH_D03G0112
GhSRS18	GH_D05G0727
GhSRS19	GH_D05G1925
GhSRS20	GH_D06G0043
GhSRS21	GH_D07G1295
GhSRS22	GH_D08G0142
GhSRS23	GH_D09G1211
GhSRS24	GH_D10G2135
GhSRS25	GH_D11G1906
GhSRS26	GH_D13G0303
GaSRS1	Gar02G20170
GaSRS2	Gar03G02410
GaSRS3	Gar03G17500
GaSRS4	Gar05G07410
GaSRS5	Gar05G19780
GaSRS6	Gar06G00710
GaSRS7	Gar06G17730
GaSRS8	Gar07G14820
GaSRS9	Gar08G01540
GaSRS10	Gar09G14350
GaSRS11	Gar10G24070
GaSRS12	Gar11G20520
GaSRS13	Gar13G06540
GaSRS14	GarUnG12180
AtSRS1	AT1G19790.1
AtSRS2	AT1G75520.1
AtSRS3	AT2G18120.1
AtSRS4	AT2G21400.1
AtSRS5	AT3G51060.1
AtSRS6	AT3G54430.1
AtSRS7	AT4G36260.1
AtSRS8	AT5G12330.1
AtSRS9	AT5G33210.1
AtSRS10	AT5G66350.1
ZmSRS1	Zm00001d032040_T001.RefGen_V4
ZmSRS2	Zm00001d006209_T001.RefGen_V4
ZmSRS3	Zm00001d053208_T001.RefGen_V4
ZmSRS4	Zm00001d014762_T001.RefGen_V4

ZmSRS5	Zm00001d036426_T001.RefGen_V4
ZmSRS6	Zm00001d038081_T001.RefGen_V4
ZmSRS7	Zm00001d021290_T001.RefGen_V4
ZmSRS8	Zm00001d021285_T001.RefGen_V4
ZmSRS9	Zm00001d009971_T001.RefGen_V4
ZmSRS10	Zm00001d011843_T001.RefGen_V4
CaSRS1	transcript:Tc01v2_t002160.1
CaSRS2	transcript:Tc02v2_t006280.1
CaSRS3	transcript:Tc09v2_t020710.1
CaSRS4	transcript:Tc08v2_t002990.1
CaSRS5	transcript:Tc10v2_t011980.1
SmSRS1	409925.v1.91
SmSRS2	59082.v1.91
SmSRS3	59069.v1.91
SmSRS4	59269.v1.91
OsSRS1	LOC_Os01g72490.1.MSUv7.0
OsSRS2	LOC_Os05g32070.1.MSUv7.0
OsSRS3	LOC_Os06g49830.1.MSUv7.0
OsSRS4	LOC_Os08g43410.1.MSUv7.0
OsSRS5	LOC_Os09g36160.1.MSUv7.0
PpSRS1	PAC:32915118
PpSRS2	PAC:32982176

Supplementary Table 2. Primers for *GhSRS21* expression and binary construction

Name of primers	Primer sequences (from 5' to 3')
GhSRS21-CDS-F	acgggggacgagctcggtaccATGGGGATGGTTGGTCTCCG
GhSRS21-CDS-R	gcccttgctcaccatgtcgacGTTTATTGGATTACCATAAGTTGAACCACC
GhSRS21-RT-F	CAAGATTGTGGGAACCAGGCCA
GhSRS21-RT-R	CAAGTCTCGGCTTCTTAGCCCC
GhUBQ7-RT-F	GAAGGCATTCCACCTGACCAAC
GhUBQ7-RT-R	CTTGACCTTCTTCTTCTTGTGCTTG
GhSRS21-VIGS-F	gtgagtaaggttaccgaattcGTCGTAACAAACTCAGTGGGA
GhSRS21-VIGS-R	gagacgcgtgagctcggtaccCCAGCACAGGCAGTAGTAGG

Supplementary Table 3. The characteristic information of *GhSRS* genes

Gene name	Locus_ID	Chromo-some	Location	Strand	Protein Length (aa)	pI	Molecular Weight (Da)	RING-like zinc-finger (aa) domain (aa)	Subcellular Location
GhSRS1	GH_A01G1110	At01	23112612-23113927	+	307	6.08	33885.55	92-240	Nucleus
GhSRS2	GH_A02G1950	At02	107486913-107487989	+	283	6.58	30790.88	89-220	Nucleus
GhSRS3	GH_A03G0939	At03	26673475-26674207	-	195	5.55	22116.24	82-193	Nucleus/Cytoplasm
GhSRS4	GH_A03G2224	At03	110047776-110048549	-	214	8.83	23519.38	22-148	Nucleus
GhSRS5	GH_A05G0731	At05	6573583-6574722	-	342	8.8	36219.08	133-293	Nucleus
GhSRS6	GH_A05G1887	At05	17853571-17854986	+	310	8.49	34126.76	88-244	Nucleus
GhSRS7	GH_A06G0057	At06	445200-446333	+	308	7.55	34107.8	85-242	Nucleus/Cell wall
GhSRS8	GH_A07G1312	At07	22785052-22786244	-	345	8.51	35799.57	137-291	Nucleus
GhSRS9	GH_A08G0136	At08	1044927-1046047	+	338	8.56	34886.71	127-284	Nucleus/Chloroplast
GhSRS10	GH_A09G1252	At09	66911518-66912662	+	357	8.05	37005.75	140-296	Nucleus/Cell membrane
GhSRS11	GH_A10G2026	At10	103297049-103298273	+	279	6.52	29951.17	86-238	Nucleus
GhSRS12	GH_A11G1874	At11	24465208-24466730	-	342	5.97	36680.26	115-258	Nucleus/Cell membrane
GhSRS13	GH_A13G0304	At13	3491606-3492355	+	204	9.21	23133.12	13-134	Nucleus
GhSRS14	GH_D01G1151	Dt01	17425941-17427261	-	309	6.51	34286.96	94-242	Nucleus
GhSRS15	GH_D02G0996	Dt02	21317312-21318074	-	205	6.3	23302.68	82-203	Nucleus
GhSRS16	GH_D02G2394	Dt02	68313518-68314294	-	214	8.75	23485.26	22-148	Nucleus
GhSRS17	GH_D03G0112	Dt03	787943-789032	-	287	5.87	31235.31	91-224	Nucleus
GhSRS18	GH_D05G0727	Dt05	5828178-5829314	-	340	8.8	35880.8	133-291	Nucleus
GhSRS19	GH_D05G1925	Dt05	16401262-16402696	+	315	8.54	34751.35	88-249	Nucleus
GhSRS20	GH_D06G0043	Dt06	322068-323199	+	308	8.33	34093.92	85-242	Nucleus/Cell wall
GhSRS21	GH_D07G1295	Dt07	17643893-17645086	-	345	8.32	35952.75	137-292	Nucleus/Cell membrane
GhSRS22	GH_D08G0142	Dt08	1081284-1082692	+	434	8.76	45074.19	223-380	Cell membrane
GhSRS23	GH_D09G1211	Dt09	38118130-38119273	+	357	7.66	36993.67	140-296	Nucleus
GhSRS24	GH_D10G2135	Dt10	56121738-56122959	+	279	6.52	29965.24	86-238	Nucleus
GhSRS25	GH_D11G1906	Dt11	20575264-20576785	-	342	5.49	36510.99	115-258	Nucleus/Cell membrane
GhSRS26	GH_D13G0303	D13	2817417-2818149	+	202	9.22	22758.74	13-134	Nucleus

[1] Chromosome: start position-end position; [2] strands: (-) means antisense strand of chromosome, (+) means positive-sense strand of chromosome; [3] RING-like zinc-finge domain: DUF702, Domain of unknown function (PF05142)
